# Point-of-care testing in community pharmacies to improve antimicrobial stewardship in respiratory infections: a scoping review of effectiveness, implementation and cost-effectiveness

**DOI:** 10.1093/jacamr/dlag106

**Published:** 2026-06-16

**Authors:** Sajal K Saha, Shukla Promite, Carly L Botheras, Elizabeth Manias, Nomvuyo Mothobi, Suzanne Robinson, Eugene Athan

**Affiliations:** Centre for Innovation in Infectious Disease and Immunology Research (CIIDIR), Institute for Mental and Physical Health and Clinical Translation (IMPACT), School of Medicine, Deakin University, Geelong, Victoria, Australia; National Centre for Antimicrobial Stewardship, The University of Melbourne, Melbourne, Victoria, Australia; Great Southern Bank, Werribe, Melbourne, Victoria, Australia; Centre for Innovation in Infectious Disease and Immunology Research (CIIDIR), Institute for Mental and Physical Health and Clinical Translation (IMPACT), School of Medicine, Deakin University, Geelong, Victoria, Australia; Monash Nursing and Midwifery, Faculty of Medicine, Nursing and Health Sciences, Monash University, Clayton, Victoria, Australia; AMR-One Health Working Group, Burnet Institute, Melbourne, Victoria, Australia; Deakin Health Economics, Institute for Health Transformation, Deakin University,Geelong, Victoria, Australia; EnAble Institute, Curtin University, Perth, Western Australia, Australia; Centre for Innovation in Infectious Disease and Immunology Research (CIIDIR), Institute for Mental and Physical Health and Clinical Translation (IMPACT), School of Medicine, Deakin University, Geelong, Victoria, Australia; Deakin Clinical Trials Hub, Deakin University, Geelong, Victoria, Australia

## Abstract

**Objective:**

To explore evidence on effectiveness, feasibility, cost-effectiveness and implementation factors of point-of-care testing (PoCT) in community pharmacies for antimicrobial stewardship in respiratory tract infections (RTIs).

**Methodology:**

A systematic scoping review of PoCT studies published between 1 January 2012 and 30 July 2025 included randomized and non-randomized trials, health economic evaluations, post-implementation surveys and qualitative studies. Study quality was assessed using critical appraisal tools. Quantitative outcomes were summarized descriptively, and implementation factors were mapped using the Consolidated Framework for Implementation Research.

**Results:**

Twenty-seven of 2689 studies were included; all but one were from high-income countries. Non-comparative studies suggested that PoCT reduced antibiotic supply. In C-reactive protein (CRP)-PoCT studies (*n* = 3; 503 patients), a median of 79.5% (IQR 72.2%–85.4%) had CRP <20 mg/L, indicating no need for antibiotics, while 1.2% (IQR 0%–11.3%) had CRP >100 mg/L, suggesting antibiotics were required; 12% were referred to general practitioners. In Group A *Streptococcus* (GAS)-PoCT studies (*n* = 12; 53 918 patients), 24.9% (IQR 17.8%–28.5%) tested positive and 21.3% (IQR 16.8%–27%) received antibiotics. In influenza-PoCT studies (*n* = 5; 374 patients), 34% (IQR 19.8%–36.1%) tested positive and 25.5% (IQR 10.1%–31.8%) received antivirals. Limited health economic evidence suggested that GAS-PoCT may be cost saving or cost minimizing. Thirteen interventions were identified to improve PoCT adoption.

**Conclusion:**

PoCT is feasible in community pharmacies to improve antimicrobial stewardship in RTIs. Successful uptake requires context-specific strategies, such as pharmacist training, collaboration with general practitioners and the implementation of appropriate reimbursement models. High-quality randomized trials are needed to confirm effectiveness and cost-effectiveness of pharmacy-led PoCT for policy implementation.

## Background

Antimicrobial resistance (AMR) and weak primary health care systems are recognized by the World Health Organization among the leading global public health threats.^1^ Misuse of antibiotics in primary care, driven by diagnostic uncertainty of the type and severity of infection, exacerbates resistance, especially in respiratory tract infections (RTIs).^2,3^ Integration of point-of-care tests (PoCTs) into routine primary care, including community pharmacies, could play a central role in enabling rapid differentiation between viral and bacterial RTIs and guiding appropriate antibiotic use.

PoCT use in RTIs has demonstrated a positive impact on antibiotic prescribing behaviours and clinical decision-making confidence in primary care, particularly in general practice.^4^ However, access to PoCTs and their impact on establishing antibiotic stewardship in community pharmacies remain unclear. Limited access to PoCTs may further exacerbate health disparities particularly in rural and resource-limited communities.^5,6^ Community pharmacies may provide an accessible setting for PoCT-guided RTI management by addressing barriers such as geographic distance to primary care services, limited access to medical appointments and the need for stronger doctor–pharmacist collaboration to support antimicrobial stewardship.^7^

A Cochrane review of 11 cluster randomized controlled trials found that C-reactive protein point-of-care testing (CRP-PoCT) can reduce RTI-related antibiotic prescribing by general practitioners by 24%.^8^ However, comparable evidence in the community pharmacy setting remains limited. The use of Group A *Streptococcus* PoCTs (GAS-PoCTs) to effectively manage acute pharyngitis or tonsillitis by community pharmacists has been demonstrated in high-income countries.^9^ Around 70% of pharyngitis cases are unnecessarily treated with antibiotics in primary care, despite only around 20% of adult cases and 5%–15% of paediatric cases are caused by bacterial pathogen, GAS.^10,11^

Despite growing interest in the use of PoCTs to manage RTIs in community pharmacies, several uncertainties remain. These include whether pharmacies are suitable settings for delivering PoCTs, whether pharmacists have sufficient time and preparedness to provide PoCT services and how pharmacists can avoid unnecessary testing. Questions also remain from patient perspectives on whether they would accept PoCT service from pharmacists and whether patients would be willingness to pay for the service. Furthermore, the factors at health system level remain less understood to sustainably implement PoCTs service and strategies by community pharmacists.^12^ A clearer understanding of the effectiveness, feasibility, practicality, safety and cost-effectiveness of PoCTs is therefore essential to inform policies that can support its integration into routine pharmacy practice. Given the wide variation in community pharmacy practice, regulatory frameworks, healthcare policies and business models worldwide, the success of PoCT implementation for RTIs would be multifactorial and context dependent. A deeper understanding of these factors is also critical to support scalable implementation of PoCT in the community pharmacy settings.

This scoping review aims to (i) explore the range and diversity of evidence on the use of PoCT for managing RTIs in community pharmacies, (ii) assess the effectiveness, feasibility, and economic viability of using PoCT in supporting antibiotic stewardship, and (iii) explore factors influencing PoCT implementation and adoption in the community pharmacy settings.

## Methodology

Our published scoping review protocol^13^ describes the methodological details. We undertook the review using the Preferred Reporting Items for Systematic Reviews and Meta-Analyses extension for Scoping Reviews (PRISMA-ScR) checklist^14^ and Arksey and O’Malley’s framework.^15^

### Search strategy

Using a uniform search strategy as reported in the protocol,^13^ we systematically searched six databases dating from 1 January 2012 to 30 July 2025. Medical databases included Medline, Emcare, PubMed, Health Technology Assessment, Cochrane Central Register of Controlled Trials and Google Scholar. Studies published from 2012 onwards were included because the use of PoCT began gaining attention in the national and international AMR action plan at this time. Search results were exported to EndNote, and duplicates were removed before screening the abstracts. We also excluded conference abstracts and preprints. A manual search was conducted for the reference lists of studies meeting the review inclusion criteria.

### Study selection

Detailed inclusion and exclusion criteria are outlined in the published protocol.^13^ We included PoCT studies if they reported on the use, effectiveness, feasibility, cost-effectiveness or implementation in community pharmacy settings for the management of RTIs and/or support antimicrobial stewardship. RTIs included, but were not limited to, otitis media; sore throat, pharyngitis and tonsillitis; common cold; rhinosinusitis and cough or bronchitis. We included a comprehensive range of study designs, including randomized and non-randomized trials, health economic studies and qualitative studies. Eligible studies involved any implementation approaches, with or without collaboration with general practitioners. We included post-implementation mixed-method or qualitative studies assessing the implementation factors influencing PoCT adoption.

### Screening and data extraction

Two authors (S.K.S. and S.P.) independently screened the titles and abstracts, reviewed full texts and resolved conflicts through discussion. One author was contacted via email for clarification. Data extraction included study details (authors, year, country), methods (aims, design, data collection, duration), settings, population (pharmacies, patient age, infection type), PoCT characteristics, implementation models and relevant outcomes such as effectiveness, feasibility, cost-effectiveness and implementation factors. For Likert-scale survey measures, implementation factors were extracted when ≥50% of respondents agreed or strongly agreed. Quantitative studies with open-ended questions on barriers or facilitators were also included regardless of respondent agreement levels.

### Quality assessment

For quality assessment, the Critical Appraisal Skills Programme (CASP) tools were used for descriptive and observational studies,^16^ as well as for qualitative studies,^17^ while the Joanna Briggs Institute (JBI) Critical Appraisal Checklist for Economic Evaluations^18^ was applied to health economic studies (Tables S1–S3, available as Supplementary data at *JAC-AMR* Online). Two reviewers (S.K.S. and S.P.) independently appraised all included studies, with discrepancies resolved through discussion and consensus. For the CASP tools, each criterion was scored using a three-point scale (0–2), where 0 indicated the criterion was not met, 1 indicated it was partially met and 2 indicated it was fully met. For the JBI economic evaluation tool, the total number of ‘Yes’ responses across the 11 criteria was used to categorize study quality as high (>8/11), moderate (6–8/11) or low (≤5/11).

### Data collation and analysis

Data were synthesized following the Arksey and O’Malley scoping review framework. Descriptive statistics summarized quantitative outcomes on feasibility, effectiveness and cost-effectiveness for each PoCT [CRP, GAS and rapid influenza diagnostic testing (RIDT)]. Owing to heterogeneity in study designs and sample sizes, results were summarized using medians and ranges. A deductive content analysis guided by the Consolidated Framework for Implementation Research (CFIR)^19^ was used to identify barriers and facilitators to PoCT implementation. Implementation determinants were mapped across the five CFIR domains: intervention characteristics, inner setting, outer setting, characteristics of individuals and implementation process.

## Results

### Scope of evidence on the use of PoCT for managing RTIs in community pharmacies

**Figure 1.**
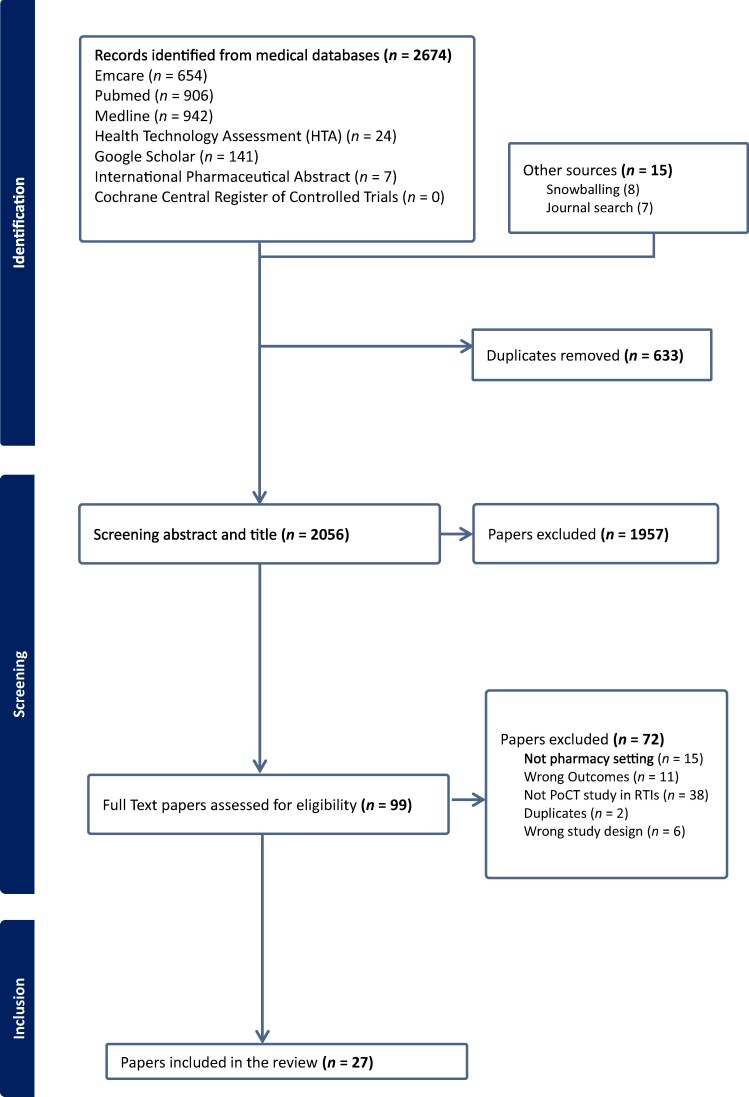
PRISMA diagram.

Our search yielded 2689 articles; 633 duplicates were removed and 2056 abstracts were screened. Ninety-nine articles underwent for full text review, with 27 papers included (Figure 1). Of 27 papers,^10,20–45^ one was a cluster randomized controlled trial on simulated patients,^23^ 18 were non-randomized non-comparative studies^10,22–37^ and two were health economic studies.^38,39^ Six other studies^40–45^ were qualitative interviews (3), survey (2) and mixed-method (1) study that reported PoCT implementation factors, barriers and facilitators from the perspective of community pharmacists and patients. Studies were related to three different PoCTs including CRP (*n* = 7), GAS (*n* = 17) and RIDT (*n* = 3). Two studies involved the use of both GAS and RIDT. PoCTs used in the studies included were TGA approved.

The studies were conducted over seven countries, predominantly high-income countries: the UK (11), USA (8), Canada (2), Australia (3), France (1), Vietnam (1) and Nigeria (1). PoCT implementation held with pharmacies located in urban (5), suburban (1), rural (1) and both urban and rural areas (14). Only one CRP study involved rural UK pharmacies. Most (66%) studies included both metropolitan and rural pharmacies. In eight studies, pharmacists independently prescribed antibiotics. In 13 studies, community pharmacists required authorization from general practitioners (GPs) for prescribing antimicrobials, and this was supported by GP–pharmacist collaborative practice agreement models.

### Study quality (critical appraisal)

Regarding the quality of 18 feasibility or observational studies, CASP scores ranged from 8 to 20 out of possible 20 points, with 12 rated as high quality (score 16–20), 4 as moderate quality (score 10–15) and 2 as low quality (score <10) (Table S1). The post-implementation studies (*n* = 6) involving qualitative outcomes (*n* = 6) were generally found high quality; CASP scores ranged from 16 to 20, out of a possible 20 points (Table S2). Out of three studies reporting health economic measures, one was of moderate quality and other two were of low quality (Table S3).

### PoCT effectiveness in pharmacy

We found no randomized controlled trials or comparative studies that assessed PoCT’s effectiveness in reducing antibiotic use or antibiotic supply in RTIs or improving guideline-adherence of antibiotic use.

### Effect of CRP-PoCT on antibiotic use based on non-comparative studies

In three non-comparative feasibility trials,^20–22^ we observed low antibiotic requirement for RTI patients who were tested for CRP level (Table 1). Out of 503 patients tested, a median 79.5% (IQR 72.2%–85.4%) with CRP <20 mg/L indicated no need for antibiotics, <1.2% (IQR 0%–11.3%) with CRP level >100 mg/L indicated a need for antibiotic, and 14.5% (IQR 9%–26%) with grey-zone CRP levels of 20–100 mg/L suggested delayed antibiotic prescribing or no antibiotic prescribing (Table 1). One study^22^ found that provision of CRP-PoCT in the pharmacy reduced RTI-related GP appointments with likely reduced chance of unnecessary antibiotic prescribing.

**Table 1.** Effect of PoCT on guiding antibiotic use from non-comparative studies

PoCT type	Total no. of patients tested (number of studies)	Test outcome (%) [median (IQR)]	Percentage of received antibiotic prescription [median (IQR)] (%)	Percentage of test positive received antibiotic treatment
CRP	503 (*n* = 3)	CRP level >100 mg/L: 1.2 (IQR 0–11.3)	1.2 (0–11.3) (Indicated for antibiotic prescription)	—
		CRP level 20–100 mg/L: 14.5 (9–26)	14.5 (9–26) (indicated for delayed antibiotic prescription)	—
		CRP level <20 mg/L: 79.5 (72.2–85.4)	79.5 (72.2–85.4) (Not indicated for antibiotic use)	—
GAS	53 918 (*n* = 12)	24.85 (IQR 17.8–28.45) test positive	21.3 (IQR 16.8–27) received antibiotics	96.1 (95.4–100)
RIDT	374 (*n* = 5)	34 (IQR: 19.8–36.1) test positive	25.5 (10.05–31.75) received antiviral	73.7 (range 40–92.3)

There was only one cluster randomized trial^23^ using simulated clients to test whether CRP-PoCT training and its access in community pharmacy can reduce non-prescription antibiotic dispensing in RTIs. Antibiotic dispensing was significantly decreased by 15.66%.^23^ However, the study did not include simulated RTI patients who directly requested antibiotic prescriptions. The Hawthorne effect was uncertain in this trial as pharmacy staff had not anticipated the client visits. CRP test results (viral, bacterial or indeterminate) were not explicitly analysed. As the simulated clients were healthy adults, CRP levels were expected to be <30 mg/L, indicating no need for antibiotics. Antibiotics were not dispensed in 43.75% of visits where CRP testing was performed.

### Effect of GAS-PoCT on antibiotic use based on non-comparative studies

Out of 53 918 patients tested (*n* = 12),^10,24–34^ median 24.85% (IQR 17.8%–28.45%) were GAS positive and median 21.3% (IQR 16.8%–27%) received antibiotic treatment (Table 1). Overall, the results from the non-comparative studies showed low antibiotic use for patients visiting pharmacies with sore throat or pharyngitis infections and who were tested for GAS. One of the trials^26^ found that the service in pharmacy was associated with reduction in phenoxymethylpenicillin prescribing by 0.4% (−3.8% and −3.4%) in primary care compared with areas where there was no GAS-PoCT service available. However, the overall reduction of the pharmacy supply of antibiotics was not different. Evidence from a UK policy experiment^32^ comparing two clinical pathways—pharmacy management of acute sore throat with and without PoCT—found that antibiotics were supplied in 72.7% of cases (95% CI: 72.5%–72.8%) under the pharmacy-first policy without testing, compared with 29.9% (95% CI: 29.4%–30.5%) when PoCT was included, suggesting a likely reduction in unnecessary antibiotic use.

### Effect of RIDT PoCT on antiviral use

In five feasibility and pilot studies, 374 patients were screened in 61 pharmacies for influenza testing. We found that out of 374 patients (*n* = 5),^29,34–37^ median 34% (IQR: 19.8%–36.1%) were influenza A positive, 25.5% (10.05%–31.75%) received antivirals (Table 1). Of those tested positive, a median 73.7% (range 40%–92.3%) received antiviral medication (Table 1).

### Feasibility of PoCT services in pharmacy

Overall, PoCT services were found feasible to implement in the community pharmacies. PoCT(s) implemented were well-received by both pharmacists and patients and both groups believed that the test would improve care for respiratory infections.

### CRP-PoCT feasibility

Three studies^20,21,22^ show that CRP-PoCT in community pharmacies is feasible, well accepted, and useful for triage of RTI patients (Table 2A). The studies together recruited 503 patients (aged 18–65 years) and pharmacists tested an average of 168 patients per study over 3–6 months, managing >88% of cases independently, with ∼12% referred to GPs. Overall service uptake as reported in the studies was 28.1%, covering ∼21% of eligible RTI patients.

**Table 2.** Feasibility of PoCTs use in the management of RTIs in pharmacy by type of PoCT (2A: CRP-PoCT; 2B: GAS-PoCT and 2C: RIDT PoCT)

Author [ref.]	Country and participating pharmacies (*n*)	Study design	PoCT type, name and source	Service duration (month)	Antibiotic Prescribing model	Patient age (years)	Patient included	Patient tested (*n*) (%) Service uptake (%)	Test outcome *n* (%)	GP referral [*n* (%)]	Self-management [*n* (%)]	Conclusion
2A: CRP-PoCT Programme												
Sim *et al.* 2021 [20]	Australia *n* = 5	Feasibility study	CRP	3 m	Collaborative (GP referral)	18–65	131	131 (100) (28)	>100 mg/L: *n* = 0 (0) 20–100 mg/L: *n* = 19(14.5) < 20 mg/L: *n* = 112 (85.4)	15 (11.5)	125 (95.4)	PoCT-CRP testing was a feasible and well accepted in community pharmacy as a triage point for RTI management
Wakeman *et al.* 2018 [21]	UK *n* = 1	Feasibility study	CRP	6 m	Collaborative (GP referral)	16–75	52	44 (84.6) -	>100 mg/L: *n* = 5 (11.3) 20–100 mg/L: *n* = 4 (9) < 20 mg/L: *n* = 35 (79.5)	6 (13.6)	33 (75)	PoCT- CRP testing has the potential to reduce RTI-related GP appointments and therefore, may help reduce unnecessary antibiotic prescribing.
O’Neill *et al.* 2022 [22]	UK *n* = 17	Observational pilot study	CRP	5 m	Collaborative (GP referral)	18–65	328	328 (100) (−)	>100 mg/L: *n* = 4(1.2) 20–100 mg/L: *n* = 85 (26) < 20 mg/L: *n* = 237(72.2)	38 (12)	38 (12)	PoCT-CRP testing service was successful for assessment of non-pneumonic lower RTIs and both stakeholders and patients reported positive experiences.
Onwunduba *et al.* 2023 [23]	Nigeria *n* = 10	RCT	CRP	6 m	Independent		600	300 (21.6)	CRP <30 mg/L: no antibiotic supply 15.66 reduction of antibiotic supply	Not reported	100	Observed uptake of CRP testing suggested that CRP testing was feasible in pharmacy.

2B: GAS-PoCT programme														
Author	Country and pharmacies (*n*)	design	Test name	Service duration (months)	Antibiotic prescribing model	Patient age	Patient consulted/included	Patient tested (*n*) (%)	Tested positive % (*n*)	Percentage of test positives received Abx	Percentage tested received Abx(*n*)	GP referral (%)	Pharmacists self- management (%)	Conclusion
Mantzourani *et al.* 2023 [24]	UK *n* = 134	Cross-sectional study	GAS (RADT)	3 m	Independent	12–44 Y	11 304	8666 (76.6)	28.9 (2503)	96.1 (*n* = 2405)	21.3 (2406)	9.3	91	GAS-PoCT-RADT service can be delivered in pharmacy at scale by ensuring appropriate use of testing and antibiotics. The service could substantially reduce workload from a common illness in other heavily pressurized areas of primary care
Mantzourani *et al.* 2022 [25]	UK *n* = 81	Follow-up study	GAS (RADT)	3 m	Independent	16–75 Y	4468	3369 (75.4)	31 (1044)	94.6 (*n* = 987)	21 (940)	NR	NR	Pharmacy data suggested that for every 100 sore throat patient consultations with a Centor score of ≥3 or a FeverPAIN score of ≥2, PoCT use may avoid up to 36 courses of antibiotics and up to 47 for patients with higher clinical scores
Mantzourani *et al.* 2020 [26]	UK *n* = 56	Pilot study	GAS (RADT)	5 m	Independent	>6 Y	1725	1239 (71.8)	28 (350)	97.1 (*n* = 27)	27.4 (340)	13.4	85	5 months GAS-PoCT service suggested balancing uncomplicated sore throat management from general practice to community pharmacies for antibiotic stewardship.
Thornley *et al.* 2016 [27]	UK *n* = 35	Feasibility study	GAS (RADT)	4–7 m	Independent	>12Y	367	149 (40.5)	24.2 (36)	100 (*n* = 36)	9.8 (36)	7.3	91	Feasible to deliver a community pharmacy to promote antibiotic stewardship
Papastergiou *et al.* 2018 [10]	Canada *n* = 204	Feasibility study	GAS (RADT)	6 m	Collaborative	5–14 Y	—	7050	25.5 (1797)	68.7 (*n* = 1234)	17.5 (1234)	NR	NR	Results highlighted public readiness to access GAS-PoCT services in community pharmacies and pharmacists’ ability to expedite management of patients with GAS including timely physician referral and appropriate antibiotic prescription
Demore *et al.* 2018 [28]	France *n* = 98	Feasibility study	GAS (RADT)	6 m	Collaborative	>18 Y	556	336 (60.4)	8.3 (28)	100 (*n* = 28)	8.3 (28)	2.7	96.5	GAS-PoCT service was feasible and could increase adherence to guidelines
Kirby *et al.* 2020 [29]	USA *n* = 2	Observational study	GAS (RADT)	5 m	collaborative	>5 Y	—	35	22.8 (8)	100 (*n* = 8)	22.8 (8)	8.3	91	Pharmacists established an effective collaboration with a primary care provider and testing was considered as a convenient service that pharmacists can offer
Klepser *et al.* 2016 [30]	USA *n* = 55	Pilot study	GAS (RADT)	10 m	Collaborative	18–45 Y	316	273 (86.3)	17.6 (48)	95.8 (46)	16.8 (44)	—	100	Pharmacists demonstrated their ability to provide pharmacist-physician collaborative pharyngitis care with avoiding overuse of antibiotics
Klepser *et al.* 2019 [34]	USA *n* = 2	Feasibility study	GAS (RADT)	18 m	Collaborative	≥5 Y	—	89	18 (16)	100 (16)	18 (16)	4.4	95.6	Pharmacists were able to offer appropriate treatment to patients without overuse of antibiotics using a protocol-driven disease management programme
Mantzourani *et al.* 2024 [31]	UK	cross-sectional study	GAS (RADT)	15 m	Independent	—	27 441	20 763 (75.6)	33 (6833)	95.4 (6519)	24	8.7	91	Despite a dramatic increase in sore throat consultation rates, pharmacists ensured appropriate use of antibiotics, and absorbed a substantial workload that would otherwise end up in other healthcare settings
Matuluko *et al.* 2024 [32]	UK	Descriptive study	GAS (RADT)	6 m	Independent	—	27 684	12 107 (43.7)	27 (3365)	96 (3231)	29.9	NR	NR	PoCT could lead to reductions in unnecessary antibiotic supply.
Edokpayi *et al.* 2025 [33]	UK *n* = 29	cross-sectional study	GAS (RADT)	6 m	Independent	>16 Y	59	36 (61)	14 (12)	—	—	NR	NR	Rapid diagnostic testing for Strep A, combined with FeverPAIN screening, could prevent antimicrobial misuse, alleviate NHS pressure and empower pharmacists.

2C: RIDT PoCT programme														
Author	Country and pharmacies	Design	PoCT Test name	Service duration (Month)	Antiviral prescribing model	Patient age	Patient consultation	Patient tested (*n*)	Tested positive percentage, % (*n*)	Percentage of test positives received antiviral	Percentage of tested patients received antiviral, % (*n*)	GP referral (%)	Pharmacist self- management (%)	Conclusion
Klepser *et al.* 2019 [34]	USA *n* = 2	Feasibility study	RIDT	18 m	Collaborative	≥5 Y	202	159 (78.7%)	38 60	85 (51)	32	4.4	95.6	Pharmacists were able to timely provide influenza treatment using physician–pharmacist collaborative care model
Kirby *et al.* 2020 [29]	USA *n* = 2	Observational study	RIDT	5 m	Collaborative	>5 Y	—	38	34.2 (13)	92.3 (*n* = 12)	31.5	8.3	91	RIDT testing service highlights the accessibility and convenience of community pharmacists. Pharmacist can fill in being extenders of health care testing and treatment, especially in populations that lack adequate insurance coverage or are not established with a primary care provider if expanded to more states.
Klepser *et al.* 2016 [35]	USA *n* = 55	Pilot study	RIDT	8 m	Collaborative	21 to 85 years (median 34 years)	121	75 (61.9%)	11 (8)	62.5 (5)	6.6	3	97	Using an evidence-based collaborative practice agreement with primary care, pharmacists were able to provide timely treatment to patients with and without influenza.
Papastergiou *et al.* 2026 [37]	Canada *n* = 2	Feasibility study	RIDT	2.7 m	Collaborative	5 and older	—	59	34 (*n* = 20)	40 (8)	13.5	NR	NR	Results highlight the readiness of community pharmacists to participate in the management of patients with influenza and their ability to implement screening into pharmacy workflow. Timely physician communication remains a barrier to access to the treatment.
Hardin 2020 USA [36]	USA *n* = 31	Pilot Implementation study	RIDT	34 days	Collaborative		—	43	28.6 (*n* = 12)	91.6 (11)	25.5	NR	NR	Implementing protocol-driven influenza services can have a significant impact in community pharmacy settings. Patients were willing to pay for the service out of pocket, showcasing the need for pharmacy-based PoCT testing

The table describes the study design, test use, prescribing model, antibiotic use, patient referrals and self-management on testing and key conclusion of the studies.

RADT, rapid antigen detection testing; NR, not reported; Abx, antibiotic.

In the UK, pharmacists delivered advice to 50% of patients on self-care, while in Australia, 95% received self-care advice. Linked GP–pharmacy models in the UK^21^ and Ireland^22^ facilitated patient referrals for testing, with higher CRP levels associated with more referrals and OTC use. The Irish pilot^22^ involved 17 pharmacies connected to nine GPs, with 60% of tested patients in pharmacies were referred by GPs and 38% of patients visited pharmacies independently. Post-CRP-PoCT service, 12%–13.6% of patients were immediately referred by pharmacists to GPs, mainly due to high-risk patients, persistent symptoms or severe illness.^21,22^ Studies consistently highlighted that enhanced GP–pharmacy collaboration may play a critical role for programme feasibility. ^21,22^

Three studies^20–22^ evaluated patient experiences and perceptions regarding CRP-PoCT services. In the Australian study,^20^ >50% (58/114) of patients had changed their perceptions regarding the necessity for antibiotics to treat RTIs and 14.3% (16/112) intended to seek a GP prescription. Patients were highly satisfied (100%) with the service, demonstrated willingness (93.4%, 123/131) to use the service again and held beliefs (92.3%; 120/130) that CRP-PoCT may enhance collaborations between doctors and pharmacists. Patients in the survey reported that the pharmacists’ advice and recommendations were very clear (93.2%). The Ireland study^22^ surveyed 89 (27%) of 328 tested patients and found similar results. Overall, 99% of participants were ‘very satisfied’ or ‘satisfied’ with the service. In addition, 98% found pharmacists’ advice as helpful. Most patients (88%) reported that the test reassured them about the reasons of cough. Furthermore, 96% were ‘very likely’ or ‘likely’ to use the service again. Similarly, 97% were ‘very likely’ or ‘likely’ to recommend the service to their friends or family. Interestingly, 66% of patients were referred by GPs to the pharmacy to receive this service. The UK study^21^ found that 95% of tested patients reported a satisfactory experience who would have otherwise visited the GP with expecting an antibiotic prescription. Of the 38 patients who were categorized by the test results to ‘watch and wait’ or self-care, none subsequently revisited the pharmacy or sought an appointment at their GP surgery.

### Feasibility of implementing GAS-PoCT in pharmacy

The GAS-PoCT was also found feasible for routine implementation in the community pharmacies, based on 12 studies^10,24–34^ (Table 2B). A total of 53 918 patients were successfully tested over an average of 5.6 months. All studies confirmed pharmacists’ capacities to safely deliver the PoCT service and guided treatment, while one^30^ indicated public readiness for GAS-PoCT. Several studies^10,27,35^ demonstrated feasibility even outside normal pharmacy hours, aiming to improve patient access to GAS-PoCT. In five studies,^10,28,29,30,34^ the physician–pharmacist collaborative practice agreement and collaborative care model for GAS-PoCT implementation was reported as critical for implementation success and sustainability, which will ensure patient safety (Table 2B). the Mentzourani *et al.*^24^ study in the UK showed that a significant number of patients were referred by GPs to the pharmacy for GAS-PoCT (*n* = 5580, 49.4%), along with self-referral by patients (*n* = 5267, 46.6%).

In another UK study,^26^ 51.9% (*n* = 896) of GAS tested patients gave consent to receive a follow-up phone call and pharmacists completed 537 follow-up phone calls. Most [91.6% (492/537)] felt completely or mostly better after receiving the PoCT service and treatment from the pharmacists, with 15.1% (*n* = 81) contacted another healthcare professional after testing. The study found no increase in the incidence of quinsy. In another UK study,^42^ 98% of 510 patients were satisfied with the GAS-PoCT service. The service satisfaction and patients’ confidence to manage RTI conditions were not dependent on whether patients were supplied antibiotics. Almost all respondents reported that they would return to pharmacy for subsequent services.

### Feasibility of implementing RIDT in pharmacy

A total of 374 patients were successfully tested over an average of 6.5 months (2.7–18 months) in five studies. Pharmacist managed >90% of patients tested, with a median 4.4% (3%–8.3%) of GP referrals. From five studies, it was found that pharmacists could provide protocol-driven timely treatment to patients with and without influenza provided that collaborative practice agreements with primary care providers is ensured to establish required communication with physicians (Table 2C).

### Cost-effectiveness of the GAS-PoCT service

Table 3 describes health economic evaluations of the reported studies. Four studies^27,33,38,39^ reported cost analysis of GAS-PoCT rather than cost–benefit or utility assessments such as cost per quality adjusted life year. None of these studies used randomized controlled trial designs or included societal costs. The US study^38^ identified GAS-PoCT as a cost-minimizing strategy, and the Canadian study^39^ identified it as cost saving when compared with physician care across five provinces. The analysis of the Canadian study showed that the mean costs per patient ranged from $37.55 to $61.57 for GPs and $38.88 to $57.56 for walk-in clinics while in pharmacies, the cost was $19.12 to $21.83. With pharmacy-based GAS-PoCT services, the cost savings per patient ranged from $12.47 to $24.36, with total annual savings estimated between $1.3 million and $2.6 million across the five provinces. The figures only accounted for direct costs and excluded indirect savings such as improved access and reduced unnecessary antibiotic prescriptions. Two observational studies^27,33^ in the UK estimated cost savings using avoided GP consultations when GAS-PoCT was available in pharmacies: £2747 per annum in one study^27^ without including service delivery costs. The other study^33^ estimated substantially higher savings, ranging from £215 205 477 in 2018/2019 to £263 211 361 in 2023/2024. The cost savings per participant were £21.54 per GP visit avoided while considered pharmacy test costs per participant, £9.46 and the average standard GP consultation cost £31.26.^33^ However, this estimation was based on a very small sample size. Of the 36 participants tested, 34 reported that they would have visited their GP. Among these participants, 11 tested positive. This implied that 67% (23/34) of GP consultations were avoided.

**Table 3.** Cost-effectiveness evidence of PoCTs service in pharmacy

Author and year	Country	PoCT	Methods	Mean cost per patient for pharmacy/family care	Mean cost savings per patient	Approximate cost savings	Threshold analysis most cost-minimizing option
Klepser *et al.* 2012 [38]	USA	GAS	A cost-effectiveness analysis	$53.56/$88.97	—	—	<$55.56 Max charge for GAS testing would be $72.74 ($55.56 for pharmacist cost and $17.18 for RADT test cost)
Lathia *et al.* 2018 [39]	Canada (5 provinces)	GAS	cost-minimization analyses	$19.12 to $21.83 compared to physician care $37.55 to $61.57	$12.47 to $24.36 per patient	$1.3 to $2.6 million per year	At a false negative probability of >0.22, physician observation is the cost-minimizing option at $80.40 followed by physician culture at a false negative probability of >0.24 and a cost of $82.30
Thornley *et al.* 2016 [27]	UK	GAS	Estimated from avoided GP visits	—	—	£2747 (from avoiding GP consultation without considering service delivery costs)	—
Edokpayi *et al.* 2025 [33]	UK	GAS	Estimated from avoided GP visits	—		Estimated total cost savings from avoided GP visits per year for sore throat: £215 205 477 (2018/2019 GBP) £263 211 361 (2023/2024 GBP)	

RADT, rapid antigen detection testing.

### Factors driving PoCT implementation in community pharmacy

PoCT implementation barriers and facilitators were analysed using reported outcomes of studies derived from pharmacist interviews^40,41,43,45^ focus groups with pharmacists,^43,45^ patient surveys,^10,20,28,29,42,44^ telephone follow up with patient tested,^21^ quantitative descriptive measures^27,30,35,37^ and mixed-method evaluation^41,45^ (Table S4). Pharmacists and patients both had practical experience of using identified PoCTs. Data were categorized into 58 implementation factors under five domains of the Consolidated Framework for Implementation Research (CFIR) framework (Table 4). These factors were mapped onto 10 of the 38 CFIR constructs. Thirteen interventions were identified against 58 implementation factors to improve the PoCT adoption and antibiotic stewardship in community pharmacy (Table 4).

**Table 4.** Key PoCT Implementation factors identified through CFIR mapping and intervention strategies to address the barriers and increase adoption of the testing in routine pharmacy

CFIR domains	Barriers	Facilitators	Intervention strategies
Intervention characteristics (11 factors)	Early stage of disease may not be reflected in CRP testing Heavy documentation Billing for PoCTs in pharmacy Difficulty while follow-up patients Cost of the test	Quick and easy to use Convenient duration for doing the testing Convenience for patients Patient reassurance with a low CRP test result PoCT aids appropriate clinical decision for antibiotic use Help to priorities patients who need referrals	Promoting user-friendly feasible and cost-efficient test Test access and availability in pharmacy Quick guide about the PoCT analyser Extra stuff to support PoCT use Working protocol for PoCT use Patient communication aids Providing decision-making aids Establishing a private clinical care area in pharmacies for PoCT Rights to prescribe antibiotics upon PoCT Use PocT use quality control framework GP–pharmacy collaborative practice agreement for PoCT use Policy supporting PoCT use by pharmacist Establishing reimbursement for pharmacy PoCT service and Medicare support Marketing and advertisement regarding the service Pharmacist training on clinical assessment of patient Pharmacist training on PoCT use
Inner settings (13 factors)	Competing demand Pharmacist time constraints Inadequate pharmacy stuff Increased workload Lack of protocol Limited provision of clinical service Lack of space/privacy for patient consultation	Sufficient protocol to guide patient management Patient flyer to raise awareness of the service More stuff Training to improve confidence of using the testing Motivation to expand the scope of practice Financial benefits for pharmacy	
Outer settings (21 factors)	Lack of reimbursement (Inadequate remuneration to justify multiple pharmacists at one time) Lack of GP–pharmacist collaboration Challenging interactions with general practitioners No community marketing campaign Refusal from patients (lack of time/service awareness, needling phobia (CRP) and unwillingness Relevant communication with doctors Legislative policy for PoCT use	Enhanced relationships with GPs Pharmacist’s prescribing authority for antibiotics Routine introduction of PoCT into daily practice Support from the national and international pharmacy organization for advanced community pharmacy services Legislative action and policy support for services involving skin penetration (finger prick blood testing) Remuneration to cover the costs of service provision Opportunity for professional development Consumer acceptance of PoCT service Marketing and promotion to encourage service uptake Patients’ satisfaction and positive attitudes towards testing Willing to pay for the service out of pocket by patients, indicating the usefulness of pharmacy-based PoCT testing Willingness for the service uptake again Changes of patient perceptions about their need for antibiotics Improving public awareness on reducing inappropriate antibiotic use and unnecessary visits to GPs or hospital emergency department	
Characteristics of individual (9 factors)	Lack of awareness of testing Perceived lack of need	Credibility of pharmacy service Belief that the service was an opportunity for pharmacists to implement AMS and to fight against antimicrobial resistance Pharmacist belief that the service would rebalance primary care resources Belief that PoCT testing benefit community Public demand View that PoCT testing is feasible and valuable Comfort in providing service by pharmacist	
Process (4 factors)	Insufficient education in pharmacy curriculum about PoCT service	On-site training and related documentation User-friendly documentation Public education about pharmacist role	

### CFIR domain 1: intervention characteristics

#### Complexity

Community pharmacists generally found PoCT to be simple, reliable, fast and accurate.^10,20,27,40,41^ These features facilitated its integration into pharmacy workflows.

#### Adaptability and cost

Adaptability and task delegation facilitated PoCT implementation. Delegating technical tasks to pharmacy technicians helped save pharmacists’ time. However, several barriers were identified. These included increased costs for patients and difficulties with patient follow up, which is not a standard practice in pharmacies.^24,40,41,43,45^

#### Relative advantage

Pharmacists perceived PoCT as beneficial for clinical decision making, especially in determining the need for antibiotics, and valued its role in enhancing patient access and collaboration with physicians.^20,40,41,43,29,35,43^

### CFIR domain 2: inner settings

#### Available resources

Limited resources hindered the implementation of PoCT in routine pharmacy practice. Key barriers included limited time, staff shortages, increased workload, workflow limitations and a lack of standardized documentation.^40,41^

#### Organizational incentives and rewards

Incentives and opportunities to expand pharmacy services encouraged the implementation of GAS-PoCT and CRP-PoCT in community pharmacies. Pharmacists were motivated by the potential to enhance their clinical role, improve patient care and increase the value of pharmacy services. Financial benefits and professional recognition also supported service adoption and continuation.^20,40,41^

### CFIR domain 3: outer settings

#### External policy and incentives

The primary external barrier was the absence of reimbursement for PoCT services.^20,40,43,45^ Facilitators included legislative support,^25,39,41,43^ reimbursement availability^24,41,43^ and prescribing authority^27,43,41^ for pharmacists.

#### Interprofessional collaboration

Collaboration with GPs was often impeded by communication barriers such as unwillingness of GP-receptionists to allow pharmacists to speak with the GP, with suggestions for secure messaging systems such as GPs’ contact email addresses to improve interactions.^21,33,40,41,43^

### CFIR domain 4: process

Promotion strategies,^40,41,42,44^ such as public education and advocacy by pharmacy organizations, were identified as enablers to attract customers and expand testing services.

### CFIR domain 5: characteristics of individual

#### Knowledge and beliefs about the intervention

Pharmacists viewed testing as a valuable clinical service, recognizing public demand and the potential benefits for patient care.^10,24,25,26,43,45^

#### Individual stage of change

##### Pharmacist level

Pharmacists’ willingness to adopt PoCT was noted as an enabler. However, a lack of awareness among pharmacists and patients remained a barrier.^40,43^

##### Patient level

Patients generally reported high satisfaction rates, appreciating the convenience and professionalism of testing services provided by pharmacists.^20,24–27^ In countries such as Australia and Ireland, >90% of surveyed patients found services helpful and expressed willingness for reuse and recommendations for other patients.^20,24^ Many patients felt reassured and avoided unnecessary GP visits, with minimal adverse outcomes reported.

Patients’ willingness to pay for the service varied, with some patients willing to pay more post-service, while others expressed financial constraints.^20,24,25^ The Australian and US studies investigated patients’ willingness to pay for the CRP-PoCT service.^20,44^ The study in Australia^20^ found that 84.6% (99/133) of patients were more willing to pay for CRP-PoCT at Day 5 follow-up than to pay immediately after the service (61.8%) (81/131). Of the 99 participants willing to pay at Day 5, >40% indicated that they would pay AUD 5–10 (41.4%), AUD 15–20 (41.4%) and AUD 25–50 (17.2%). By contrast, those who refused to pay for the service perceived that the government or a third party should bear this service cost. In the Vietnam study,^45^ >78% (407/520) of participants expressed willingness to pay for CRP-PoCT in pharmacies: 36% were willing to pay >USD 2.20% and 5.4% were willing to pay >USD 5.40.

In a US study, some patients refused to pay for the service because of financial constraints. Others believed they could distinguish between mild and serious illness on their own. Limited encouragement and support from healthcare professionals, mainly GPs, also reduced patients’ willingness to use community pharmacy testing.^29^ Lack of health insurance coverage for testing costs prevented patients from using the service.^29^ The cost of care was important to 58 (93%) of patients to determine where they sought GAS testing in pharmacy; 57 (92%) would be more likely to come to the pharmacy if the cost were less than the cost of visiting a doctor’s office. Around 62% of patients tested were willing to pay $50 or more for pharmacy provided GAS management.^30,44^

## Discussion

We synthesized findings from 27 studies to report effectiveness, feasibility, cost-effectiveness and implementation factors which will guide future policies and research on PoCT use in the community pharmacy settings. Evidence is still lacking to conclude the effect size and cost-effectiveness of PoCT in pharmacy to reduce antibiotic use in RTIs. The type of PoCT(s) that were found in the review (CRP/GAS/RIDT) are individually feasible to implement in community pharmacy. However, there are some important factors that need to be considered to improve adoption of the testing into routine pharmacy care. These include pharmacists’ training on patient clinical assessment, test availability, patient-facing resources, physician–pharmacist collaborative practice agreement for PoCT use, reimbursement for the new service, patients’ satisfaction and willingness to pay for the service, and appropriate diagnostic stewardship approach.

From non-comparative studies, we found low use of antibiotics for the tested patients in pharmacies. However, we identified no studies that directly measured the effect size of reducing overall antibiotic supply or on antibiotic prescribing by GPs in primary care. This evidence gap highlights the need for future trials to measure the impact on real-world settings. Future trials can be designed in the contexts where GP and pharmacy are co-located and distantly located for a deeper understanding of the implementation mechanisms, along with effectiveness.

The GP–pharmacist collaborative care model was used in a few reviewed studies^22,29,30,35^ to facilitate PoCT implementation and antibiotic prescribing in pharmacies. The collaborative model could increase patient safety and avoid unnecessary testing in community pharmacy. The use of CRP-PoCT, where the primary concern is the diagnostic accuracy, as in some patients, elevated CRP could be caused by various reasons, including viral infections or protozoal infections.^46^ Six studies showed that collaborative care models for PoCT use would be more feasible if some issues were resolved, such as timely interactions between GPs and pharmacists regarding the test results and prescribing by establishing a GP–pharmacy collaborative practice agreement.

However, GPs’ unwillingness to engage and the fear of the strain of interprofessional relationship^43,47^ is reported as factors influencing collaboration and the provision of PoCT in pharmacies. The interprofessional conflict and the lack of legal frameworks and treatment guidelines are found as barriers to CRP-PoCT implementation in Vietnam.^45^ In many countries such as Singapore, the United Arab Emirates (UAE), Hong Kong, Sudan, Pakistan, Bangladesh and India, pharmacists are viewed primarily as dispensers rather than healthcare providers. Pharmacies are also often perceived as business-oriented services to doctors and patients in Japan, Singapore, Jordan, UAE and Russia.^48^ Furthermore, prescribing authority for pharmacists may support independent PoCT implementation, as suggested in the UK and by some French pharmacists.^43,47^ In a systematic review of Wu *et al.*^49^ community pharmacist-led antimicrobial supply was found to be associated with reduced unnecessary antibiotic prescribing for pharyngitis, low levels of re-treatment, adverse events and improvement of subsequent healthcare use and high rates of clinical improvement of patients treated.

Strengthening the role of community pharmacists as primary care providers may support wider adoption of PoCT for antimicrobial stewardship. Key enablers include legal frameworks, clinical assessment training, regulatory guidance, structured referral pathways, GP–pharmacist communication and GP awareness of pharmacy testing services, which are aligned with other reviews.^50,51^ Local laws and regulations vary and should be considered when implementing PoCT. As services expand, patient awareness and the value of testing can be demonstrated at scale. A systematic review^52^ reported that pharmacists in the USA, Wales, Australia and France perceive themselves as accessible and well-positioned to use CRP and GAS-PoCT to reduce GPs’ workload.

This scoping review found consistent positive patient experiences on pharmacy-based PoCT services, including both CRP-PoCT and GAS-PoCT. Across settings and countries, patients reported high satisfaction, strong willingness to reuse the testing and recommending the service to others, regardless of whether antibiotics were supplied. Importantly, PoCT appeared to support appropriate self-care in RTIs without increasing subsequent healthcare use or complications. In our review, patient willingness to pay for PoCT services emerged as an important facilitator. Willingness to pay varied, with greater acceptance observed when costs were modest or lower than GP visits, and when services were supported by insurance or public funding. Collectively, these findings suggest that pharmacy-led PoCT services are acceptable to patients and may enhance antimicrobial stewardship while maintaining high-quality patient-centred care.

However, an important additional consideration for pharmacy-based PoCT implementation is the need for appropriate diagnostic stewardship. Easier access to PoCT through pharmacy could increase expectations for diagnostic testing in situations where self-care would normally be sufficient. This may lead to greater use of PoCT for mild or self-limiting RTIs, potentially increasing healthcare use without corresponding clinical benefit. Such patterns could affect the long-term cost-effectiveness of pharmacy-based PoCT services, especially in publicly funded healthcare systems where unnecessary testing may place more pressure on limited healthcare resources. To support sustainable implementation, PoCT services may require clear clinical criteria for performing the testing, structured pharmacist assessment processes and patient education on self-management and appropriate use of PoCT. These measures may help ensure that PoCT is directed towards patients most likely to benefit while avoiding unnecessary or low-value use of PoCT.

In reality, to support the routine use of PoCT in pharmacies, affordability is of major concern for patients, the health system and national policy makers. We found no evidence from existing studies that evaluate the cost-effectiveness of CRP-PoCT in pharmacy. There were two studies that estimated the cost savings from a pharmacist-provided GAS-PoCT testing and treatment service. However, it was unclear how and whether a GAS-PoCT service fits into a pharmacy business model in the different countries in general. As the PoCT service takes time, practically, this new service can be implemented sustainably if sufficient staff are available and the profit made in pharmacy is equal to or greater than profit gained from dispensing prescriptions. A time motion study in Australia found that pharmacists spent much time on dispensing (30%), indirect patient services (17%), counselling (15%), supply and management activities (15%), and little time for providing more professional services (15%).^53^ This indicates the likely necessity of an extra supporting pharmacist(s) to facilitate PoCT services in future.

In our review, pharmacy PoCT implementation in the USA, Canada and the UK was found as cost saving or a cost-minimizing option from the perspective of public funding. However, the main limitation is that those studies were not focused on cost-effectiveness. Although studies showed cost savings, outcomes could be worse or better, which needs future investigations. The International Pharmaceutical Federation (FIP) recognizes that PoCT service in the pharmacy could have benefits for both publicly funded and insurance-funded healthcare systems.^54^ This review highlights the need for future research to evaluate the cost-effectiveness of GAS-PoCT services in countries considering implementation, with a particular focus on paediatric populations in which GAS infections are more prevalent than in adults. Further investigation is also warranted in high-risk settings, such as Indigenous Australian communities where the burden of GAS infection and rheumatic heart disease remains disproportionately high.^55,56^

### Implications for research, policy and practice

Implementation of PoCT in community pharmacy should consider resource requirements, patient outcomes, antimicrobial resistance and healthcare delivery costs. Currently, evidence is insufficient to support definitive policy recommendations, highlighting the need for establishing effectiveness and cost-effectiveness evidence of available PoCT(s).

Further theory-informed qualitative research, along with well-designed randomized controlled trials are required to better understand PoCT implementation, particularly in rural community pharmacies and in settings with a high prevalence of acute rheumatic fever and rheumatic heart disease, such as among Indigenous Australians.^56^ In these contexts, improved access to GAS-PoCT may support earlier diagnosis and prevention of serious complications.

Strengthening collaborative relationships between GPs and community pharmacists is likely to be critical for sustained and successful PoCT implementation in pharmacies. However, research that explores viable strategies to build a strong, trustworthy and respectful interprofessional relationship is required. Overall, the findings of this review may support health service researchers, stakeholders and policymakers in developing PoCT implementation strategies as part of the broader antimicrobial stewardship programmes that aim to improve the care and management of respiratory infections in primary care.

### Strengths and limitations

To the best of our knowledge, this is the first comprehensive scoping review outlining existing evidence on the effectiveness, feasibility and cost-effectiveness of PoCT in the management of RTIs in community pharmacy. This review excluded surveys and qualitative studies that were not embedded within PoCT implementation trials or involved participants without direct experience of PoCT use in routine pharmacy practice. As a result, our findings reflect experienced rather than perceived barriers and facilitators. Both quantitative and qualitative data were synthesized using the theory-informed CFIR to describe key implementation factors.

Although we proposed a search period from 1 January 2012 to 31 December 2022 in the published protocol,^13^ we conducted search until 30 July 2025 to ensure the review included the most recent and relevant evidence without altering the review objectives or eligibility criteria. We excluded any PoCT studies targeted at COVID-19 purposefully because COVID-19 testing is implemented primarily for public health surveillance rather than for guiding antimicrobial stewardship in RTIs. The limited number of randomized controlled trials prevented meta-analysis of the outcomes of interest. Heterogeneity of reported outcomes across studies prevented meta-analytic presentation of review outcome measures. Finally, variations in pharmacy practices and policies across countries may limit the generalizability of the findings.

### Conclusions

There is emerging evidence that PoCTs are feasible to implement in community pharmacies with patient satisfaction to support RTI management and antibiotic stewardship. Randomized controlled trials and research investments are required both in high-income and low-income countries to comprehensively assess the effectiveness and cost-effectiveness of PoCT(s) for policy decisions. Contextual multifaceted strategies are critical to foster implementation and adoption of testing into routine pharmacies for enhanced antibiotic stewardship in RTIs.

## Supplementary Material

dlag106_Supplementary_Data

## Patient and public involvement

Patients and/or the public were involved in the design, conduct, reporting or dissemination plans of this research. Patients were not directly involved in our research, but the studies included in this review may include patient populations.

## Supplementary data

Tables S1–S4 are available as Supplementary data at *JAC-AMR* Online.
