# Supplementary material for: Point-of-care testing in community pharmacies to improve antimicrobial stewardship in respiratory infections: a scoping review of effectiveness, implementation and cost-effectiveness
Source: JAC Antimicrob Resist. 2026 Jun 16;8(3):dlag106. doi: 10.1093/jacamr/dlag106 (PMC13270238; doi:10.1093/jacamr/dlag106)
Supplement: dlag106_Supplementary_Data [file dlag106_supplementary_data.docx]

**Supplementary Appendix**

**Table S1**: Quality appraisal of descriptive and observational studies using the CASP tool

| **Authors** | a clearly focused issue addressed? | method  appropriate? | recruitment  acceptable? | measures accurately measured? | data collected appropriately? | participant enough for reliable estimates? | Results accurate? | data analysis rigorous? | clear statement of findings? | results be applied to the local  population? | **Total out of 20** |
| --- | --- | --- | --- | --- | --- | --- | --- | --- | --- | --- | --- |
| Sim et al 2021 [20] | 2 | 2 | 2 | 2 | 1 | 2 | 2 | 2 | 2 | 2 | 19 |
| Wakeman et al 2018 [21] | 2 | 2 | 1 | 2 | 2 | 1 | 2 | 1 | 2 | 2 | 17 |
| O’Neill et al 2022 [22] | 2 | 2 | 2 | 2 | 2 | 1 | 2 | 1 | 2 | 2 | 18 |
| Onwunduba et al 2023[23] | 2 | 2 | 2 | 0 | 1 | 1 | 2 | 1 | 2 | 1 | 14 |
| Mantzourani et al 2023[26] | 2 | 2 | 2 | 2 | 2 | 2 | 2 | 2 | 2 | 2 | 20 |
| Mantzourani et al 2022[27] | 2 | 2 | 2 | 0 | 2 | 1 | 2 | 1 | 2 | 2 | 16 |
| Mantzourani et al 2020[28] | 2 | 2 | 0 | 2 | 2 | 1 | 2 | 1 | 2 | 2 | 16 |
| Thornley et al 2016[29] | 2 | 2 | 2 | 2 | 2 | 1 | 2 | 2 | 2 | 2 | 19 |
| Papastergiou et al 2018[30] | 2 | 2 | 0 | 2 | 1 | 1 | 2 | 2 | 2 | 1 | 15 |
| Demore et al 2018[31] | 2 | 2 | 2 | 2 | 2 | 1 | 2 | 1 | 2 | 2 | 18 |
| Kirby et al 2020[32] | 2 | 2 | 1 | 2 | 2 | 1 | 2 | 1 | 2 | 2 | 17 |
| Klepser et al 2016[33] | 2 | 2 | 0 | 2 | 2 | 1 | 2 | 1 | 2 | 1 | 15 |
| Klepser et al 2019[34] | 2 | 2 | 0 | 2 | 2 | 1 | 2 | 0 | 2 | 2 | 15 |
| Mantzourani et al 2024 | 2 | 2 | 2 | 2 | 2 | 1 | 2 | 1 | 2 | 2 | 18 |
| Matuluko et al 2024 | 2 | 2 | 1 | 2 | 2 | 1 | 2 | 1 | 2 | 1 | 16 |
| Edokpayi et al 2025 | 2 | 0 | 0 | 0 | 2 | 1 | 2 | 1 | 2 | 0 | 10 |
| Papastergiou et al 2016[39] | 2 | 2 | 0 | 2 | 2 | 0 | 2 | 2 | 2 | 2 | 16 |
| Hardin et al 2020  USA [54] | 2 | 1 | 0 | 1 | 1 | 0 | 2 | 0 | 1 | 0 | 8 |

**Table S2.** Quality appraisal of qualitative studies using the CASP tool

| Authors | Clear aims | appropriate methodology | Appropriate research method | Appropriate recruitment strategy | Appropriate data collection | Considered relationship between researcher and participant | Considered ethical issues | Rigorous data analysis | Clear reporting of findings | Total out of 18 |
| --- | --- | --- | --- | --- | --- | --- | --- | --- | --- | --- |
| Nguyen et al 2024 | 2 | 2 | 2 | 2 | 2 | 1 | 2 | 2 | 2 | 17 |
| Czarniak et al. 2022 | 2 | 2 | 2 | 2 | 2 | 2 | 2 | 2 | 2 | 18 |
| Chalmers et al 2022 | 2 | 2 | 2 | 2 | 2 | 2 | 2 | 2 | 2 | 20 |
| Klepser 2015 | 2 | 2 | 2 | 2 | 2 | 1 | 2 | 1 | 2 | 16 |
| Mantzourani et al. 2019 | 2 | 2 | 2 | 2 | 2 | 1 | 2 | 1 | 2 | 16 |
| Mentzourani e al 2021 | 2 | 2 | 2 | 2 | 2 | 1 | 2 | 2 | 2 | 17 |

**Table S3.** Quality appraisal of heath economic studies

| Tools | Yes | No | Unclear | Not applicable | Klepser et al 2012 | Lathia et al 2018 | Edokpayi et al 2025 | |
| --- | --- | --- | --- | --- | --- | --- | --- | --- |
| 1. Is there a well-defined question? | □ | □ | □ | □ | Yes | Yes | Yes | |
| 1. Is there comprehensive description of alternatives? | □ | □ | □ | □ | Yes | Yes | No | |
| 1. Are all important and relevant costs and outcomes for each alternative identified? | □ | □ | □ | □ | Yes | Unclear | No | |
| 1. Has clinical effectiveness been established? | □ | □ | □ | □ | No | No | No | |
| 1. Are costs and outcomes measured accurately? | □ | □ | □ | □ | Yes | Yes | Yes | |
| 1. Are costs and outcomes valued credibly? | □ | □ | □ | □ | Yes | Yes | Yes | |
| 1. Are costs and outcomes adjusted for differential timing? | □ | □ | □ | □ | No | No | No | |
| 1. Is there an incremental analysis of costs and consequences? | □ | □ | □ | □ | Yes | No | No | |
| 1. Were sensitivity analyses conducted to investigate uncertainty in estimates of cost or consequences? | □ | □ | □ | □ | Yes | Yes | No | |
| 1. Do study results include all issues of concern to users? | □ | □ | □ | □ | No | Unclear | No | |
| 1. Are the results generalizable to the setting of interest in the review? | □ | □ | □ | □ | No | No | No | |
|  | Yes score out of 11 | | | | 7/11 | 5/11 | | 3/11 |
| Comment |  | | | | Moderate Quality | Low Quality | | Low Quality |

**S4: PoCT implementation barriers and facilitators in pharmacy from the perspective of pharmacists and patients**

| **Author, Year, Country** | **Aim** | **Sources of data** | **Participants (Age, Gender)** | **PoCT Type** | **Barriers** | **Facilitators** |
| --- | --- | --- | --- | --- | --- | --- |
| Czarniak et al 2022 [40]  Australia | Exploring pharmacists’ experiences  barriers and facilitators regarding the implementation and sustainability of POC CRP testing in RTI management in Western Australia | Semi- Structured Interviews | 10 Pharmacists  mean age: 36 yrs and female (80%) | CRP | -Challenging interactions with general practitioners.  -Competing demands.  -Difficulty in follow-ups.  -Early stage of disease may not be reflected in CRP testing.  -Heavy documentation.  -Inadequate remuneration to justify multiple pharmacists at one time.  -Perspectives of patients  – bulk billing from general practitioners. | -Accessibility and credibility of pharmacists.  -Enhanced relationships with general practitioners.  -Improved professional image and strengthened existing or established new pharmacist-patient relationships.  -Marketing and promotion to encourage service uptake.  -Practice and experience promote confidence.  -Supportive team. |
| Chalmers et al 2022 [41]  Australia | Mapping the implementation methodology for the pilot study of point-of-care C-reactive protein (CRP) testing to support pharmacists’ management of respiratory tract infections in Western Australian pharmacies | Mixed- Methods | 10 Pharmacists and  5 pharmacy Assistants  /Interns  Pharmacists: 33.5 yrs. 80% F  Assistants/ interns: 24 yrs. 100% F for | CRP | - | - Support from the national and international pharmacy organization for advanced community pharmacy services - Legislative action and policy support for services involving skin penetration (finger prick blood testing). - Consumer acceptance POCT service - On-site training and related documentation - User-friendly documentation - To integrate the service into existing workflows for RTI presentations, - Patient flyer to raise awareness of the service - Remuneration to cover the costs of service provision - Use of a reliable, user-friendly POC CRP testing device - Training to improve knowledge, confidence, and competence to perform CRP testing. - Appropriate remuneration for service provision. - Pharmacists believe it is feasible to provide CRP testing. - CRP testing will enhance collaboration with GPs. - Integrating efficiently with work practices. - Improving professional satisfaction as a pharmacist. - Appropriate remuneration for service provision. - Pharmacists believe that there is a public demand for testing to inform AB use, improve awareness of antibiotics, and to support triage while managing patient with RTI. - POCT assist in pharmacist’s clinical decision-making on patient referral to a GPs for antibiotics. |
| Sim et al 2021[20]  Australia | Evaluating the feasibility of POC  CRP testing to support pharmacists’  management of RTIs in community pharmacies in Western Australia. | -Survey | 131 Patients  Mean: 39.6  52.4% F | CRP | Barriers to service provision  -Refusal from patients because of patients’ lack of time and understanding of the  service,  needling phobia  unwillingness  -Pharmacy’s time constraints, and competing demands  -unavailability of a study  pharmacist.  -No community marketing campaign | -Changes of patient perceptions about their need for antibiotics.  -Improving public awareness by reducing inappropriate antibiotic use and unnecessary visits to GPs or hospital emergency department.  -Public demand for testing in community pharmacies to know he needs for antibiotics for cough and colds.  -May enhance doctors-pharmacist collaboration.   - CRP value may reinforce   pharmacists’ recommendations.  Patient satisfaction   - Willingness for the service uptake again.   - quick and easy.  -Very comfortable with the pharmacists performing the test.  -Testing should be offered in a community pharmacy.  - 84.6% (99/133) of patients indicated a willingness to pay for testing at Day 5 follow-up, which was higher than the willingness to pay immediately post-service (61.8%) (81/131). |
| Kirby et al  2020 [29]  USA | Implementing an influenza and streptococcus POCT service to expand both access to pharmacist-provided services and the clinical offerings of the pharmacy. | Survey | 73 Patients | Strep A (GAS) and Influenza | Billing for POCTs in community pharmacy that potentially prevented some patients from using the service. | -Patients satisfaction (ranged from 4.88 to 5.00 out of 5  -98% of participants reported being satisfied‚ or very satisfied  -Patients were highly likely to recommend the service to others.  -Of patients presenting for the service, majority were insured,  -Having a collaborative practice agreement that allows for lab test and prescribing   - pharmacy leadership in providing the service to benefit both the community and the pharmacy |
| Hardin et al  2020 [36]  USA | Describing the development and implementation of an influenza POC testing service in a large community pharmacy chain and identifying successes and barriers to the Implementation. | Retrospective Chart Review | 42 Patients  53% Female | Influenza | -Health insurance cannot be billed due to current pharmacy reimbursement practices. | -Willing to pay for the service out of pocket by patients, indicating the usefulness of pharmacy-based POCT testing. |
| Mantzourani et al 2019 [43]  UK | Exploring the views and opinions of community pharmacists regarding their initial experience of and levels of preparedness for the new sore throat test and treat service in Wales. | Semi-structured interviews | 7 Pharmacists  29% F | Strep A (GAS) |  | - The technology infrastructure was crucial to ensuring the service’s success.  - Pharmacists perceived the service as contributing to AMS.  - Pharmacists believed the service would rebalance primary care resources.  - Service Set-Up as A Tool for Patient Education:  -Pharmacists believed POCT was vital to decision-making and reduced unnecessary antibiotic use which adversely impact on the fight against AMR.  -POCT result supported patient education; results made patients more accepting of the consultation outcome.  - Service was an opportunity for pharmacists to implement AMS and contribute to the fight against  antimicrobial resistance. |
| Mantzourani et al 2020 [42]  UK | Understanding the impact of a new pharmacy sore throat test and treat service on patient experience | -Survey | 510 Patients | Strep A (GAS) | -Inappropriate referrals  -lack of awareness of the service  - Inconsistent commissioning and -logistical challenges | - professionalism in providing service - pharmacy staff - pharmacy local environment and system - consumer satisfaction - Perceived value |
| Wakeman et al 2018 [21]  UK | Point-of-care C-reactive protein testing in community pharmacy to deliver appropriate interventions in respiratory tract infections | - Telephone follow-up | Patients (44) | CRP  CRP | None | - All patients reported a satisfactory - experience with the quality of the consultation process and intervention - In total, 95% of patients who received the POC CRP test reported that they would have otherwise visited the GP and would have expected to be prescribed antibiotics - GP awareness of the usefulness proper interpretation of the result - Very high-test results helped provide staff with confidence in their recommendation for patients visiting a GP and seek consideration of antibiotic treatment. - A low CRP test result was found to be reassuring for the patient. |
| Papastergiou et al 2018 [10]  Canada | Assessing the implementation feasibility and effects of a community pharmacist- directed point-of-care testing program for GAS. | -Survey | Patients (1004)  Mean: 27.3 yrs  63.1% F | Strep A (GAS) | -Communication of recommendations to the physician remains a barrier. | - Speed and efficiency - Reasons for wanting to use the service again. - Fast/quick/efficient service (54%) - Access to treatment/appropriate medication sooner (13%) - No appointment necessary (6%) - 81% of patients were very or somewhat - satisfied with receiving a GAS test at the pharmacy. - 91% identified the pharmacy as a convenient location. - 93% would be very likely or somewhat likely to use the service again. - pharmacy owners and managers perceived value of the program. - Ease of implementation into the existing workflow. |
| Demore et al 2018 [28]  France | Testing the feasibility, benefit and acceptance of a community  pharmacy-based antibiotic stewardship intervention based on rapid antigen test use in adult patients with sore throats. | Survey | 559 Patients  74 Pharmacists  Median: 27.8 yrs  64.3% F | Strep A (GAS) | Patients (Most common causes for refusal)  -Lack of time to perform the test  -Low perceived benefit | -All (138/138) patients undergoing the test declared to be satisfied with the use of RAT  -Would accept the test again in the future  Pharmacists’ Feedback:  -Sufficient time spent during the training  -Easy to use the RAT  -The result was sufficient to guide clinical management  -Sufficient protocol to guide patient management  -Duration to do the testing was convenient  -Opportunity for professional development  -Would welcome the routine introduction of RAT in their daily practice, if endorsed and financed by the Health Authorities |
| Thornley et al 2016 [27]  UK | Testing the feasibility and benefit of a pilot service run from community pharmacies incorporating rapid antigen detection testing (RADT) for patients 12 years and over presenting with sore throat symptoms according to Centor criteria. | Quantitative Descriptive | 367 Patients  62% F | Strep A (GAS) |  | - Payment did not appear to be a barrier to patients   receiving the test and antibiotics as  all patients that were  eligible based on their Centor score went on to access the paid elements regardless of  deprivation index. |
| Papastergiou et al 2016 [37]  Canada | Investigating the impact and feasibility of community pharmacist- directed  influenza screening | Quantitative Descriptive | 59 Patients  Median=45  yrs  64% F | Influenza | The benefit of a rapid influenza diagnosis was lost because of the necessity for physician intervention to initiate treatment. | - Barrier could be overcome if pharmacists were granted   prescribing authority for  oseltamivir. |
| Klepser et al 2016 [30]  USA | Describing a community pharmacy-based, collaborative physician-pharmacist Group A Streptococcus (GAS) management program through characterization of the patient population and service patterns. | Quantitative Descriptive | 316 Patients | Strep A (GAS) |  | - 43.2% (118/ 273 ) of eligible - patients did not have a primary care - provider, - 43.9% (120/273) were seen at the - pharmacy outside regular clinic - office hours. - -improved access - timely care for patients   with acute illnesses.   - Physician pharmacists’ Collaborative Practice agreement assured adherence to recognized practice standards. |
| Klepser et al  2016 [35]  USA | Examining the  effectiveness of a collaborative physician- community pharmacist program to treat influenza like infection with respect to clinical outcomes and health care utilization. | Quantitative Descriptive | 121 Patients  Mean: 37 yrs | Influenza | Limited community wide influenza activity upon service offered  Turnover of trained staff during the study period. | - None |
| Klepser et al 2015 [44]  USA | Describing patient satisfaction with and willingness to pay for a community pharmacy-based GAS pharyngitis point-of-care management program. | Survey | 273 Patients | Strep A (GAS) | - | - 52 (84%) of patients were   satisfied with the care  and would go back  to the pharmacy for a similar illness in the future.   - 58 (93%) of patients indicated   that cost of care was important to them in determining where they sought care for GAS.   - 57 (92%) would be more likely to come to the pharmacy if the cost were less than the cost of visiting a doctor’s office. - (62%) were willing to pay $50 or more for pharmacy provided GAS management. |
| Vinh et al 2024  [45]  Vietnam | Exploring the acceptability and feasibility of implementing C-reactive protein point-of-care testing (CRP-PoCT) in pharmacies in Vietnam | Mixed method study | - Survey with 520 patients at 25 pharmacies  -3 focus group discussions with customers (20 participants) and  -12 in-depth interviews with  pharmacists and other stakeholders | CRP | -additional burden of  service provision  -lack of an enabling policy environment, and  -potential risks for customers | - Creating an enabling policy   Environment   - transparent discussion of values and risks - customers’ and pharmacists’ - perceived benefits of testing |
